# A simple solution for model comparison in bold imaging: the special case of reward prediction error and reward outcomes

**DOI:** 10.3389/fnins.2013.00116

**Published:** 2013-07-19

**Authors:** Burak Erdeniz, Tim Rohe, John Done, Rachael D. Seidler

**Affiliations:** ^1^School of Kinesiology, University of MichiganAnn Arbor, MI, USA; ^2^Max Planck Institute for Biological CyberneticsTuebingen, Germany; ^3^School of Psychology, University of HertfordshireHatfield, UK; ^4^Department of Psychology, University of MichiganAnn Arbor, MI, USA; ^5^Neuroscience Graduate Program, University of MichiganAnn Arbor, MI, USA

**Keywords:** prediction error, model comparison, dopamine, predicted value, fMRI

## Abstract

Conventional neuroimaging techniques provide information about condition-related changes of the BOLD (blood-oxygen-level dependent) signal, indicating only where and when the underlying cognitive processes occur. Recently, with the help of a new approach called “model-based” functional neuroimaging (fMRI), researchers are able to visualize changes in the internal variables of a time varying learning process, such as the reward prediction error or the predicted reward value of a conditional stimulus. However, despite being extremely beneficial to the imaging community in understanding the neural correlates of decision variables, a model-based approach to brain imaging data is also methodologically challenging due to the multicollinearity problem in statistical analysis. There are multiple sources of multicollinearity in functional neuroimaging including investigations of closely related variables and/or experimental designs that do not account for this. The source of multicollinearity discussed in this paper occurs due to correlation between different subjective variables that are calculated very close in time. Here, we review methodological approaches to analyzing such data by discussing the special case of separating the reward prediction error signal from reward outcomes.

## Introduction

Functional neuroimaging studies of reward and punishment learning have become an important research topic for understanding brain regions involved in decision-making and reinforcement learning (Montague et al., 2006; Rangel et al., 2008). One finding of this research is that human learning and decision-making are guided by subjective decision variables (Rangel and Hare, 2010; Bartra et al., 2013). Studies have shown that these subjective decision variables are not always directly observable by the experimenters and that computational models are needed to infer them (Corrado and Doya, 2007; O'Doherty et al., 2007; Furl and Averbeck, 2011; Mars et al., 2012). Furthermore, understanding these decision variables not only provides a framework for neuroscientists to understand where in the brain they may be calculated or represented, but it can also shed light on the possible computational mechanisms that guide efficient decision making (Gläscher and O'Doherty, 2010; Mars et al., 2012). One such decision variable is the predicted reward value of a conditional stimulus (CS) (i.e., see Gottfried et al., 2003). In order to calculate the predicted value of a CS, the reward-prediction error (RPE) associated with it should be known (Montague et al., 1996; Schultz et al., 1997). The RPE signal indicates how surprising a particular stimulus is after the organism receives the rewarding outcome associated with it. It originates from Bush and Mosteller's learning model (1951) and was later updated by the Rescorla-Wagner learning rule (1972). In its simplest form, the Rescorla-Wagner reward prediction error is calculated by the difference between the actual reward receipt (R) and the predicted reward value (*V*_*C*S_), where the RPE is represented by the symbol δ, (δ = *R* − V_*C*S_) (Glimcher, 2011).

In neuroimaging studies that use the Rescorla-Wagner form of RPE, RPE is calculated when the participants receive reward feedback (e.g., Pessiglione et al., 2006) that makes it hard to distinguish from hedonic responses to reward outcomes (RO). However, in the temporally extended versions of the RPE signal, such as the temporal-difference learning algorithm (TD), the RPE is calculated during the outcome retrieval and it shifts back to the presentation of the CS (Niv and Schoenbaum, 2008) in order to indicate an approximate prediction about the amount of the RO of the CS (e.g., O'Doherty et al., 2003). In the Rescorla-Wagner learning rule, the RPE is used to update the expectations of reward predictions for the next trial and is calculated by the following equation: *V*_*C*S, *t* + 1_ = *V*_*C*S, t_ + αδ (α indicates the stimulus specific learning rate).

Numerous electrophysiological studies in animals have reported that mid-brain dopamine neurons in the ventral tegmental area and substantia nigra perform computations that are similar to RPEs (Schultz et al., 1997; Bayer and Glimcher, 2005). Nevertheless, it has also been found that RPE activity is not limited to the mid-brain dopaminergic neurons but is also found in other parts of the brain such as the anterior cingulate cortex and medial-frontal cortex (Amiez et al., 2005; Matsumoto et al., 2007). Since the initial publication of Schultz et al. (1997), numerous brain regions have been identified that code for RPEs; this has led to different neural circuit models for prediction error (PE) coding in the brain (this suggests that PE is coded either locally in the brain or in a distributed fashion; for a review please refer to Schultz and Dickinson, 2000; Kawato and Samejima, 2007). For example, one early localist interpretation argues that the calculation of the prediction error requires that the information associated with the reward amount and the predicted value should both be available at the midbrain dopaminergic synapse in order to calculate a RPE signal (Houk et al., 1995). Since Houk et al. (1995), many neuroimaging studies have been carried out in order to identify the neural correlates of prediction error in humans (for a meta-analytic review, see Garrison et al., 2013). Furthermore, based on the economic theory, alternative axiomatic approaches have been developed to identify which brain regions are actually coding the RPE signal (Rutledge et al., 2010). The study of Rutledge et al. (2010) showed that the medial orbitofrontal cortex, striatum, amygdala and posterior cingulate cortex satisfy the necessary and sufficient condition for all classes of RPE signals. Moreover, many studies have been conducted to determine the neural correlates of a reward outcome (RO) and the predicted value (for meta-analytic reviews, see Kringelbach and Rolls, 2004; Grabenhorst and Rolls, 2011; Liu et al., 2011; Diekhof et al., 2012; Levy and Glimcher, 2012). These studies suggest that the medial orbitofrontal cortex and the striatum are the most likely candidates for brain regions that process the RPE signal, the predicted value signal, and RO. However, researchers still disagree where RPEs are coded in the brain (see Schultz and Dickinson, 2000; Garrison et al., 2013 for a discussion). One reason for this is due to its correlation with ROs.

In functional neuroimaging, determining what type of information is represented in a particular voxel is a challenging question if multiple highly correlated regressors are introduced to a general linear model (GLM; Poldrack et al., 2011). This problem of multicollinearity is not only related to poor estimation of regressors' parameter estimates, but it can also give rise to anatomical misattribution of functions if it is not taken into account (Andrade et al., 1999). In the case of RPE and RO, multicollinearity between regressors arises because both of these variables are calculated at the same time (during the time of the unconditional stimulus). In order to solve the problem of inefficient parameter estimation due to multicollinearity, many suggestions have been made by researchers such as efficient experimental design (Monti, 2011). The problem of misleading conclusions due to multicollinearity can be accounted for by rather complicated Bayesian model comparison approaches (Stephan et al., 2009). Here, we summarize an alternative and relatively simpler approach that is related to the orthogonalization of regressors within a GLM analysis.

## Solutions for model comparison

**Figure 1:**
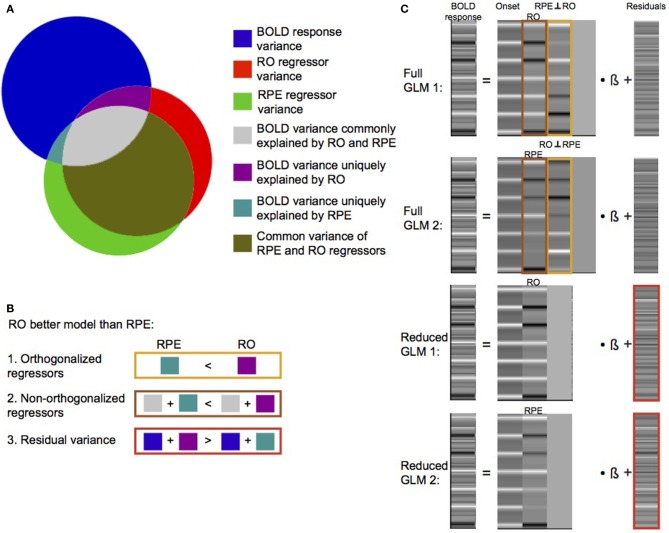
**Illustration of the three model comparison approaches. (A)** The areas of three overlapping circles correspond to unique and common variance of the dependent variable (BOLD response) and the two candidate regressors (RO vs. RPE). In this example, the RO model is a better model of the region's BOLD response than the RPE model. This can be inferred from three equivalent comparisons. **(B)** First, the comparison of BOLD variances uniquely explained by the orthogonalized regressors shows that the RO model explains more BOLD variance than the RPE model. Second, the comparison of the BOLD variances uniquely and commonly explained by the non-orthogonalized regressors leads to the same conclusion. Third, the comparison of the residual BOLD variances of the reduced GLMs comprising only one of the competing regressors shows that the inclusion of the RPE regressor leaves more residual BOLD variance than if RO is included. Thus, RO wins the model comparison also in this approach. **(C)** Four GLMs are fitted to the BOLD response. Full GLMs contain both regressors but with reversed orthogonalization. Reduced GLMs only comprise one of the competing regressors. Regressors used for the three model comparison approaches in **(B)** are color-coded.

**Figure 2:**
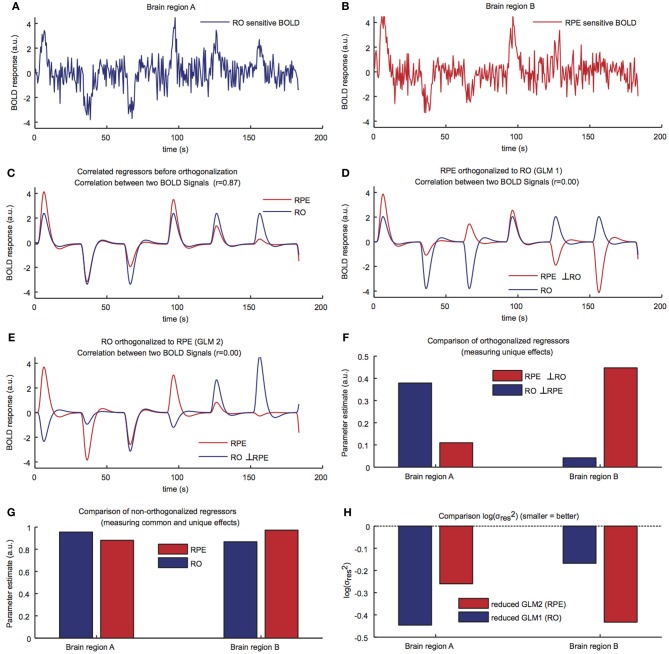
**(A,B)** A simulated BOLD signal was created for a total of 200 s for two brain regions representing mainly the RPE and the RO, respectively. Zero second duration events were used. **(C)** Correlation between the RO and the RPE regressor if there is no orthogonalization between the regressors (*r* = 0.89). **(D)** The RPE regressor is orthogonalized based on the RO regressor (*r* = 0). **(E)** The RO regressor is orthogonalized based on the RPE regressor (*r* = 0). **(F)** Parameter estimates of the non-orthogonalized regressors in the two brain regions from the two full GLMs show that RO explains more unique BOLD variance in region A and RPE explains more unique BOLD variance in region B. **(G)** Parameter estimates of the orthogonalized regressors from the two full GLMs lead to the same conclusion. **(H)** A model comparison via log residual variance (smaller = better) from reduced GLMs shows that the RO regressor provides a better fit of region A and the RPE regressor provides a better fit of region B.

Recently, using two different decision-making tasks, Rohe et al. (2012) tested where predicted values, RPE and RO are coded in the brain. As mentioned above, this is a challenging question because there is a rich source of contradictory observations; moreover, a methodological challenge arises from the fact that RPE and RO are inherently correlated (i.e., a reward results in a positive RPE and a non-reward results in a negative RPE). Consequentially, the parametric regressors, which encode the models' predictions, are highly correlated if they are both included into the general linear model. In their paper, Rohe et al. (2012) introduced three approaches to compare which of the two competing models' signals (RPE vs. RO) is a better description of a regional BOLD signal. A model comparison seeks to select the model that is better able to explain the variance of a dependent variable (e.g., a BOLD signal) while having the lowest complexity (i.e., number of free parameters) (Maxwell and Delaney, 2004). The comparison is not straightforward if the model predictions are correlated as in the case of RPE and RO. Due to the correlation, part of the variance of the BOLD signal can be equally explained by both models. However, a model comparison of correlated models with the same complexity can be implemented in three equivalent ways within a GLM approach as illustrated by the use of Venn diagrams (Figure 1). First, the model comparison can be implemented by comparing the parameter estimates assessing the BOLD variance, which is uniquely explained by the orthogonalized RO and RPE regressors (Figures 1B, 2F). Orthogonalization refers to the computational procedure that renders one regressor orthogonal to a second regressor (Rodgers et al., 1984). The non-orthogonalized regressor and the orthogonalized regressor occupy the same vector subspace as before orthogonalization, but the parameter estimates of the orthogonalized regressor now measure the BOLD variance which is uniquely explained by this regressor. To obtain parameter estimates of the orthogonalized RO and RPE regressors, two separate full GLMs (Figure 1C), each containing both model regressors but with reversed orthogonalization, are fitted to the data (Note that it is important to z standardize regressors before fitting the two GLMs because otherwise the size of parameter estimates is not only affected by the variance they can explain but also by different scaling of the models' regressors). If the parameter estimates of the orthogonalized regressors are statistically compared, the model that explains relatively more unique BOLD variance can be selected (i.e., it wins the comparison). Second, the model comparison can be implemented by comparing the parameter estimates of the non-orthogonalized regressors measuring the variance that is commonly explained by both regressors in addition to the variance uniquely explained by the regressor itself (Figures 1B, 2G). By subtraction, one effectively eliminates the variance which is commonly explained by both models. Consequently, the comparison determines which of the competing models has larger uniquely explained variance as in the previous approach. Third, the model comparison can be implemented by comparing the residual BOLD variance, which cannot be explained by the RO or the RPE regressor (Figures 1B, 2H). The better model explains more BOLD variance than the worse model. Thus, the residual variance of the BOLD signal is smaller for the winning than for the losing model. For this approach, two separate reduced GLMs are fitted, each comprising only one of the candidate models' regressors (Figure 1C). In conclusion, the details of the study determine which of the three equivalent approaches should be adopted. The third approach is the most general because it can, in principle, handle different model complexities (e.g., using Bayesian information criterion trading of model fit vs. model complexity). However, the first approach might be most feasible because it can be easily implemented in standard packages like statistical parametric mapping (SPM) (http://www.fil.ion.ucl.ac.uk/spm) using parametric regressors and SPM's inherent orthogonalization.

To further illustrate the idea of these model comparisons, we simulated a scenario in which 80% of the neurons represent an RO signal and 20% represent an RPE signal (Region A) or vice versa (Region B). In such a scenario, it is hard to differentiate the role of these regions and conclude that Region A is coding RO and Region B is coding RPE because the BOLD activations in those two regions will be highly correlated (in addition to the intrinsic correlation between RPE and RO signals).

In order to demonstrate how the three approaches to model comparison yield the appropriate model, we ran a computer simulation using Matlab (www.mathworks.com) and SPM software. The results of the simulation can be seen from the illustrative example in Figure 2. We initially generated a simulated BOLD (blood oxygenated level dependent signal) signal for two brain regions (Region A and Region B), which carried both the RO and the RPE signals (Figures 2A,B). In the simulated BOLD signal, the contribution of RO and RPE to the overall activity in Region A (RO sensitive region) was weighted as 80% RO and 20% RPE, whereas Region B (RPE sensitive region) was weighted as 20% RO and 80% RPE (plus Gaussian noise). The RPE regressor was created using a simple Rescorla-Wagner learning rule as shown in the introductory equations (α = 0.5). In modeling the simulated BOLD responses, two separate GLMs (full GLM1 and GLM2) were constructed which incorporated the RPE and the RO regressor in their design matrices (Figure 1C). Thus, GLM1 and 2 were the same except that the order of orthogonalization of the RPE and the RO regressors was reversed. Regressors were created by convolving their stimulus function with a haemodynamic response function. The stimulus function for RO was made up of the vector [1, 0, 0, 1, 1, 1, 0] (ones indicate reward delivery and zeros indicate non-delivery), which was introduced at stimulus onset times. Similarly, the stimulus function for RPE was made out of real numbers indicating the size of RPE as [1, −0.5, −0.25, 0.87, 0.43, 0.21, −0.89]. Before orthogonalization, the RO and RPE regressors were highly correlated (Figure 2C). In the first scenario, the RPE regressor was orthogonalized to the RO regressor (full GLM 1; Figure 2D) and the RO regressor was orthogonalized to the RPE regressor (full GLM 2; Figure 2E). Orthogonalization effectively reduced the correlation of regressors (*r* = 0). We then compared the parameter estimates of the orthogonalized regressors for the two brain regions (Figure 2F). The RO regressor showed higher “activation” (i.e., explained more unique BOLD variance) compared to the RPE regressor in brain Region A. Conversely, the RPE regressor showed higher activation in brain Region B. This showed that comparison of parameter estimates of orthogonalized regressors correctly identified the signal underlying a region's BOLD response. In the second scenario, we compared the parameter estimates of the non-orthogonalized regressors (Figure 2G). This approach led to significant activation compared to baseline for both models in both regions. This illustrates that if only one of the competing models' signals is investigated in isolation (i.e., only compared to baseline), this could result in a misattribution of function (e.g., we could falsely conclude that both region A and B represent RO). However, when comparing parameter estimates of the competing models, we again found that RO was a better model of region A while RPE was a better model of region B. In the final scenario, we used the same two GLMs but removed the RPE regressor from GLM 1 and removed the RO regressor from GLM 2 (Figures 1C, 2H). Next, we compared the log residual variances in order to determine which of these reduced GLMs has a better overall fit (i.e., has less residual BOLD variance). In case of a comparison of equally complex models, log residual variance can be taken to compare models because model comparison indices like Akaike or Bayesian information criterion (AIC/BIC) are a linear function of log residual variance in this case (Stephan et al., 2009). Thus, one should choose the GLM with the lowest log residual variance corresponding to minimum AIC/BIC (Pitt and Myung, 2002). Hence, we again retrieved the “ground truth” that RO is a better model of region A while RPE is a better model of region B.

Rohe et al. (2012) showed that both RPE and RO activated striatum, midbrain and the medial orbito-frontal cortex when the activation from the non-orthogonalized regressors was compared to a zero baseline. However, when the authors compared the RPE and the RO model, they showed that RO was a better model of activity in MOFC than RPE while RPE was a better model of activity in striatum and midbrain. This does not necessarily mean that these regions independently code these variables. However, they might be sharing information in order to calculate more complex variables (e.g., reward information calculated in the medial frontal cortex might be used by the ventral striatum to further calculate prediction errors).

## Summary

Rohe et al. (2012) provided evidence that RO is a better model for BOLD responses in MOFC while RPE is a better model for BOLD responses in the striatum and midbrain. However, all of these regions seemed to respond to RO and RPE if their correlation was not taken into account. Recently, more studies have begun to apply similar analysis techniques (e.g., Bornstein and Daw, 2012) and this method can be applicable to other areas of cognitive neuroscience such as numerical cognition where multicolinearity is a problem in identifying the neural correlates of parametric regressors (Wood et al., 2008). Consequently, Rohe et al. (2012) have provided a simple and elegant solution to the model comparison issue that can be applied to many experiments. Applying a comparison technique will eventually lead to the correct selection in the sense of a “ground truth” model. Finally, it is important to note that although this approach provides a practical solution for model comparison, there should be prior knowledge to explain why one of the regressors can explain the shared variance better than the other.

### Conflict of interest statement

The authors declare that the research was conducted in the absence of any commercial or financial relationships that could be construed as a potential conflict of interest.
